# Tirzepatide against obesity and insulin-resistance: pathophysiological aspects and clinical evidence

**DOI:** 10.3389/fendo.2024.1402583

**Published:** 2024-06-24

**Authors:** Salvatore Corrao, Chiara Pollicino, Dalila Maggio, Alessandra Torres, Christiano Argano

**Affiliations:** ^1^ Department of Clinical Medicine, Internal Medicine Unit, National Relevance and High Specialization Hospital Trust Azienda di Rilievo Nazionale ed Alta Specializzazione (ARNAS) Civico, Di Cristina, Benfratelli, Palermo, Italy; ^2^ Department of Health Promotion Sciences, Maternal and Infant Care, Internal Medicine and Medical Specialties. Promozione della Salute, Materno-Infantile, di Medicina Interna e Specialistica di Eccellenza (PROMISE), University of Palermo, Palermo, Italy

**Keywords:** obesity, insulin-resistance, Tirzepatide, pathophysiology, GIP, GLP - 1, clinical trials, arterial hypertension

## Abstract

Obesity is a chronic, multifactorial disease in which accumulated excess body fat has a negative impact on health. Obesity continues to rise among the general population, resulting in an epidemic that shows no significant signs of decline. It is directly involved in development of cardiometabolic diseases, ischemic coronary heart disease peripheral arterial disease, heart failure, and arterial hypertension, producing global morbidity and mortality. Mainly, abdominal obesity represents a crucial factor for cardiovascular illness and also the most frequent component of metabolic syndrome. Recent evidence showed that Tirzepatide (TZP), a new drug including both Glucagon Like Peptide 1 (GLP-1) and Glucose-dependent Insulinotropic Polypeptide (GIP) receptor agonism, is effective in subjects with type 2 diabetes (T2D), lowering body weight, fat mass and glycated hemoglobin (HbA_1c_) also in obese or overweight adults without T2D. This review discusses the pathophysiological mechanisms and clinical aspects of TZP in treating obesity.

## 1 Introduction

Obesity is a progressing and chronic condition defined by Body Mass Index (BMI) over 30 Kg/m2 levels. It is also defined as “*abnormal or excessive fat accumulation that presents a health risk*”“ (1). Rates of overweight (defined as a BMI over 25) and obesity continue to rise among the general population: between 1975 and 2016, in children and adolescents worldwide (age 5–19 years), the incidence of overweight or obesity increased by four times, from 4% to 18%. According to the WHO European report, 59% of adults and children, about 27% of girls and 29% of boys, are overweight or obese (2).

A BMI between 30 and 35 is correlated with decreased longevity by three years, and also BMI between 40 and 50 reduces lifespan by ten years confronted to people with fit BMI. Each increment in BMI of 5 kg/m^2^ greater than 22,5–25,0 kg/m^2^ corresponds to a rise in overall mortality of 30% (3), in obese people the major death cause in CVD, followed by T2D (4).

Obesity pathophysiology is multifactorial and involves social, psychological and behavioral factors, genetic and metabolic predisposition (5). Obesity occurs when energy intake is more than whole body expenditure (6). In fact, the hypothalamus, mesolimbic area and prefrontal cortex, three areas involved in executive functioning, are directly involved in controlling energy balance. Cerebral pathways correlated with appetite, satiety and energy expenditure are constantly activated (7, 8). Lastly, dysregulation of microRNAs (miRNAs) has been linked with obesity and inflammation. MicroRNAs (miRNAs) are small, RNA molecules non-coding, which regulate post-transcriptional gene expression and play an essential role in physiologic and pathologic processes. In fact, how dysregulation in miRNA production and its role in obesity phenotypes is an object of several studies (9).

Obesity is directly related to the development of cardiometabolic disease (arterial hypertension, coronaric syndrome or heart failure (10) and in particular abdominal obesity is the most frequent component of metabolic syndrome along with T2D, dyslipidemia and hypertension (11–13).

It’s also associated, in addition, with obstructive sleep apnea (OSAS), non-alcoholic fatty liver disease (NAFLD)/metabolic-associated fatty liver disease (MAFLD), osteoarthritis, gastro-esophageal reflux disease, malignancies (gastrointestinal, liver, breast, and endometrial cancer), and mental health issues, especially in young people. Insulin resistance has a critical role in the development and progression of NAFLD/MAFLD (14–16). In this sense, insulin resistance is a crucial factor shared by Obesity, T2D, and NAFLD/MAFLD.

Adipose tissue contributes to endothelial dysfunction (17) due to secretion of adipokines, paracrine hormones which have a crucial role in the regulation of vascular tone. In obesity patients, pro-inflammatory and vasoactive adipokines such as angiotensinogen, angiotensin II, aldosterone, and resisting, along with increased plasma renin activity and cytokines are hypersecreted (18–28).

Notably, some peptides termed incretins, able to stimulate β- cells to release insulin, were discovered by Barre J.L. Campo in the early 1930s. The most commonly known incretins include glucagon-like peptide-1 (GLP-1) and glucose-dependent insulinotropic polypeptide (GIP), formerly called Gastric inhibitory peptide (29, 30). The gut (31) releases these factors due to food intake and plays an essential role in appetite regulation and body weight (32), decreasing hunger and nutrient intake (32, 33), and gastric emptying and gastrointestinal motility (29, 34). In light of these effects, GLP-1 receptor agonists (GLP-1RA) can be used not only in the treatment of T2D but also to promote Weight Loss (WL) (33). Subsequently, dual GLP-1 receptor (GLP1-R) and GIP-receptor (GIP-R) agonism is able to reduce BW by more than 20% (35, 36). TZP is a new pharmacological approach which includes dual agonism, approved in May 2022 by the United States Food and Drug Administration (FDA) for the treatment of T2D and in November 2023 for the treatment of obesity (37). Acting in weight loss, it also improves quality of life and reduces obesity-related complications. Innovative pharmacological therapies and surgical approaches are valid alternatives to promote fat loss (38) to reverse the adverse effects triggered by weight gain.

In view of this, a widespread search of SCOPUS, PubMed, and CENTRAL was performed using the following string” (obesity or insulin resistance) AND (tirzepatide or Dual GIP and GLP-1 Receptor Agonist). Hand-searching for principal generalists, human nutrition and basic research journals was also done. This review aimed to analyze the knowledge available on the pathophysiological mechanisms and clinical aspects of TZP in treating obesity.

### 1.1 The first twincretin, Tirzepatide (LY3298176)

The chemical formula of TZP is C225H348N48O68, a linear synthetic peptide composed of 39 aminoacids and with a molecular weight of about 48 KDa, which arises from the “fusion” of aminoacid sequences structurally similar to glucagon, human GIP, GLP-1, exendin-4. Nineteen amino acids show similarity to those of GIP. The peptide sequence contains an Aib residue at position 2, which occupies the DPP-4 binding site, conferring DPP-4 resistance. Furthermore, it presents, at position 20, a C20 fatty acid chain attached via a linker to the lysine residue, which enables the molecule to bind circulating albumin with high affinity, thus increasing the half-life up to 5 days (39), allowing a single weekly subcutaneous administration. In receptor binding studies, it has been shown to have a GLP-1R and GLP-1 binding affinity, respectively, about five times lower than that of native and similar to endogenous GIP (40). Indeed, from a molecular perspective, its strength is comparable to native GIP but weaker than native GLP-1 (approximately 13-fold) (41). Available data suggests that hyperglycemia may cause the downregulation of GIP-R, thus reducing its response (42). However, lots of data suggest that the administration of molecules able to restore a condition of euglycemia can revert this resistance (20). Simultaneously, GIPR signaling blocks emesis and attenuates other adverse side effects of GLP-1R activation (43). Thus, TZP was introduced for the first time, to enhance the effectiveness of incretins and reduce side effects.

### 1.2 The physiology of incretins

GIP (the one which provides the most of incretin effect in humans (7)) and GLP-1, are peptide hormones secreted respectively, L cells of the bowel and K cells of duodenum after assumption of carbohydrates, triglycerides, proteins or amino acids (44, 45). GLP-1R is expressed in β-cells, in a minor population of α-cells, but also in lungs, kidneys, liver, gastric mucosa, heart, brain (in regions involved in the regulation of food intake and/or satiety) and immune cells (3–6). GIP, secreted by K cells of the duodenum and the proximal part of the small intestine, is the principal incretin hormone in humans, providing most of the incretin effect (7). GIP-R are distributed in the pancreas but also in the heart, the pituitary, the adrenal cortex, some areas of the central nervous system (CNS) and both brown and white adipose tissues where it promotes fat deposition (8). For this reason, this hormone has been assumed to promote obesity (38). Although, the function of GIP in weight management needs to be clarified. Data in the literature support the hypothesis that GIP promotes fat deposition, and *in vitro* experiments on GIP-R-deficient show a resistance to obesity (14). However, in a transgenic mouse model, persistently high GIP levels resulted in improvement of β-cell function, promote insulin sensitivity and gene transcription, glucose tolerance, and reduction in weight gain (15).

### 1.3 *In vitro* Tirzepatide’s effects

**Figure 1:**
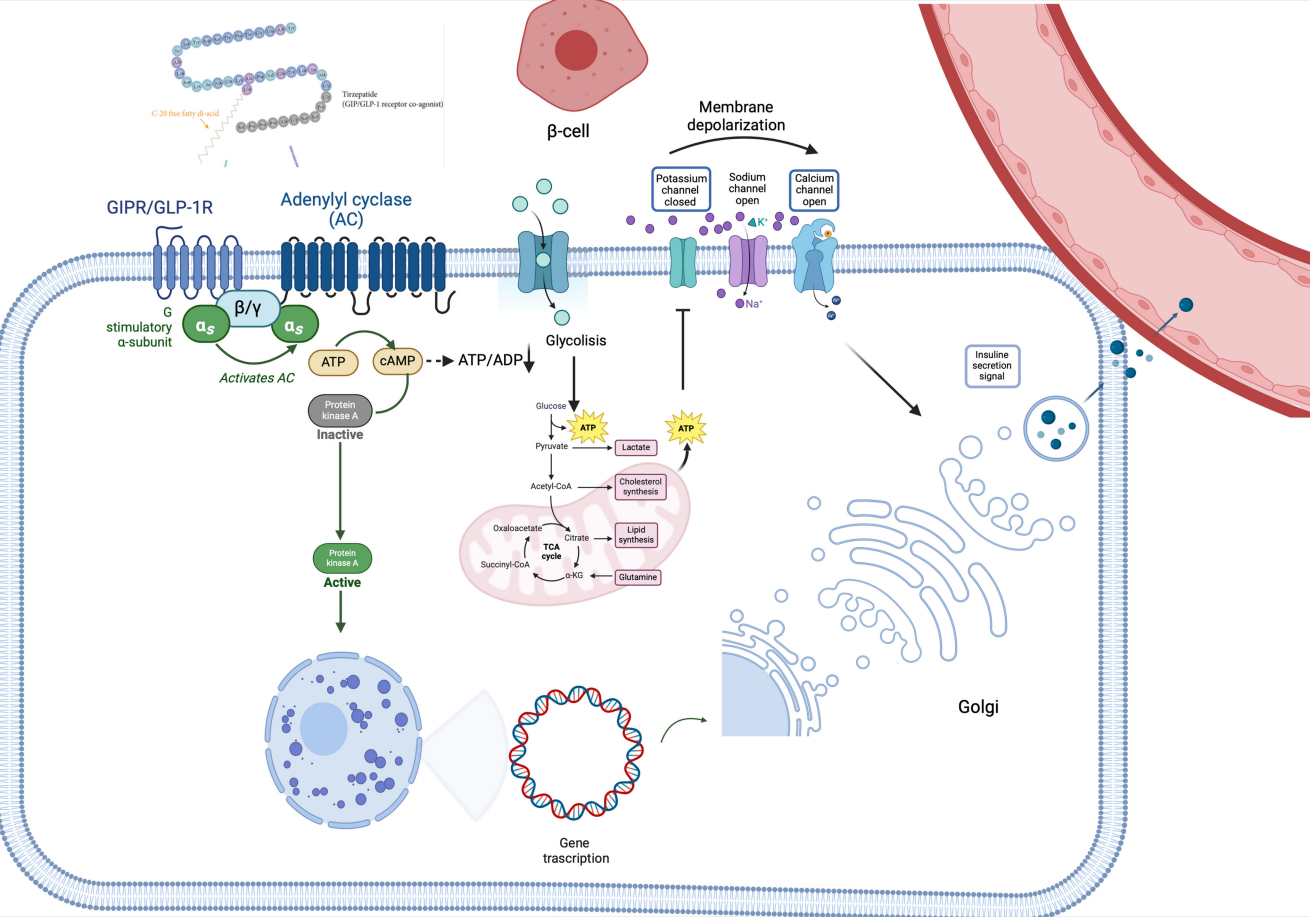
Tirzepatide’s pathway signaling. TZP binds its receptor, leading to the activation of adenylyl cyclase-cAMP-protein kinase A (PKA) pathway and thus stimulating glucose metabolism (glycolysis and Krebs Cycle). The increase of intracellular ATP levels hesitates in the closure of plasma membrane K+ channels, thus triggering β-cell depolarization. Due to depolarization, voltage-gated Ca2+ channels become open, favoring the entrance of Ca2+ into the cell, which concomitantly stimulates the releasing of calcium from the endoplasmic reticulum. This leads to the release of insulin into the bloodstream. Additionally, PKA stimulates insulin gene transcription, leading to insulin synthesis. *α_s_, in vivo-subunit; ADP, adenosine diphosphate; ATP, adenosine triphosphate; β/γ, G protein* β*/gamma subunits; cAMP, cyclic adenosine monophosphate; GIP-R/GLP-1R, gastric inhibitory polypeptide receptor/glucagon-like peptide 1 receptor; PKA, adenylyl cyclase-cAMP-protein kinase A.*

*In vivo* and *in vitro* preclinical studies have demonstrated that co-administration of GIP and GLP1 promotes insulin sensitivity, leading to better blood glucose level control, more sustained reduction in food intake and consequently better WL compared to the infusion of a single agent (35, 41, 46), also exerts an additive effect on metabolism of cyclic adenosine monophosphate (cAMP), thus increasing its levels and consequently, potentiating insulin secretion glucose-dependent. Studies conducted on rat β-cells (47) and cell lines expressing recombinant GLP-1R and GIP-R *in vitro* and human islets (41) demonstrated that the adenylate cyclase was influenced by signals arising from the stimulation of both receptors, mainly deriving from GIP (**Figure 1**). Regarding pharmacodynamics, TZP appears as a biased GLP-1R-GIPR co-agonist, because of its positive effects on cAMP generation over β-arrestin recruitment, which is conversely stimulated by GLP-1R. This results in a lower ability to promote receptor internalization compared with endogenous GLP-1, so it allows to have an increased GLP-1R expression on the cell surface, which translates in more robust insulinotropic properties, possibly explaining the enhanced insulin secretion (in both diabetic and not diabetic human islets), induced by TZP (19) by approximately 25% more than the one induced by only one of the two agents. It also improves insulin sensitivity and reduces glucagon secretion. Beyond the synergistic effects on insulin secretion and synthesis, the double agonist therapy seems to strongly promote survival and differentiation of β-cell (48) (**Figure 1**).

### 1.4 *In vivo* Tirzepatide’s effects

The above results are confirmed also in *in-vivo* studies. Co-agonism showed, on murine models, the capability to improve insulin secretion and sensitivity in a weight-dependent and weight-independent manner, probably through its action on nutrient metabolism (49, 50), preventing their accumulation and thus making these organs actively involved in metabolism with an improvement in insulin sensitivity and WL, yielding long-lasting effects. In obese mice, co-agonism is demonstrated to enhance β-cell proliferation (predominantly GLP-1), improve β-cell function (by reducing its apoptosis) and survival, and also through the reduction of glucotoxicity (49, 51, 52). The gain in insulin sensitivity could be related to the increased glucose internalization in muscle and WAT promoted by TZP, results seen in obese IR mice.

### 1.5 Other possible pleiotropic effects of Tirzepatide

Pleiotropic effects of GIP receptor stimulation in other tissues, mediated by mechanisms other than typical protein G-coupled receptor pathways, might bring additional benefits. Acting on insulin and glucagon levels, incretins, and thus TZP, may have indirect effects also on the liver and muscle (4, 6). In various models, GIP seems to stimulate LPL activity (53), modulating triglycerides (TG) release and favor its clearance (53) and deposition in white adipose tissue (WAT); also increase WAT perfusion (54–56). TZP decreases serum alanine aminotransferase and atherogenic biomarkers (chylomicrons, small dense LDL-cholesterol levels, apoB, apoC-III) (57). Relating to the presence of incretin receptors on endothelial cells, TZP can also reduce blood pressure (BP) (58). In addition to enhancing lipid metabolism, the combination GIP-GLP1 may improve weight parameters by acting on receptors located in the central nervous system (CNS). In high-fat-fed mice, the combined metabolic effect of incretins hesitates in more reduction of food intake and consequently more weight reduction (59). The reduction in food intake may be related to the activity of GIP, able to cross the blood barrier, on its receptors sited in the arcuate (ARC) nucleus and other hypothalamic centers. In CNS, GIP-R is expressed on the surface of cells both alone and in association with GLP1-R (60), acting on the same cells (facilitating GLP-1 internalization to specific neuronal populations (61)) or, as documented in other studies, on different cells (62). Interestingly, in some cells of the ARC nucleus, it is also possible to find neuropeptide receptors (molecules with a role in the regulation of calorie intake) together with GIP-R, probably implicated in the regulation of food intake. Moreover, GIP seems to attenuate GLP-1R-agonism mediated adverse effects thus making more tolerant this therapy and favoring WL. Together, this evidence suggests that GIP may drive WL directly, inhibiting caloric intake, indirectly exploiting the anorectic action of GLP-1, or by reducing GLP-1RA adverse effects (59, 60, 63). Also, it seems that there could be another mechanism involved in the reduction of food intake, probably related to the stimulation of POMC gene expression mediated by co-agonism (62). Co-treatment with GLP-1R and GIP-R agonists also results in food intake reduction and BW reduction than either agonist alone in obese mice with T2D and rats (64–66). Beyond the reduction in energy intake, co-agonism seems to be able to affect food choice, favoring the consumption of healthy nutrients, how documented in experiments on mice and rats (67). Moreover, in mice, the administration of TZP showed to inhibit gastro-enteric delay (52).

Experimental data on animal models have suggested that GIP suppresses peripheral arterial remodeling, thus showing an anti-atherosclerotic activity, by acting on receptors in the heart and vessels (19, 20). GIP appears also to reduce radical oxidative stress species (48), but also it is known the implication of GIP in the inflammatory pathways. Data suggest that activation of GIP-R decreases inflammation in adipose tissue and cytokines levels (such as IL-6) and promotes the rise of serum adiponectin levels, a cytokine with anti-inflammatory effects and beneficial effects on diabetic nephropathy, which can explain potential beneficial effects on kidneys (even if GIP-R are not present) (47). It also induces nitric oxide–mediated vasodilation and thus may have implications for CV disease (37, 68) and kidneys.

**Figure 2:**
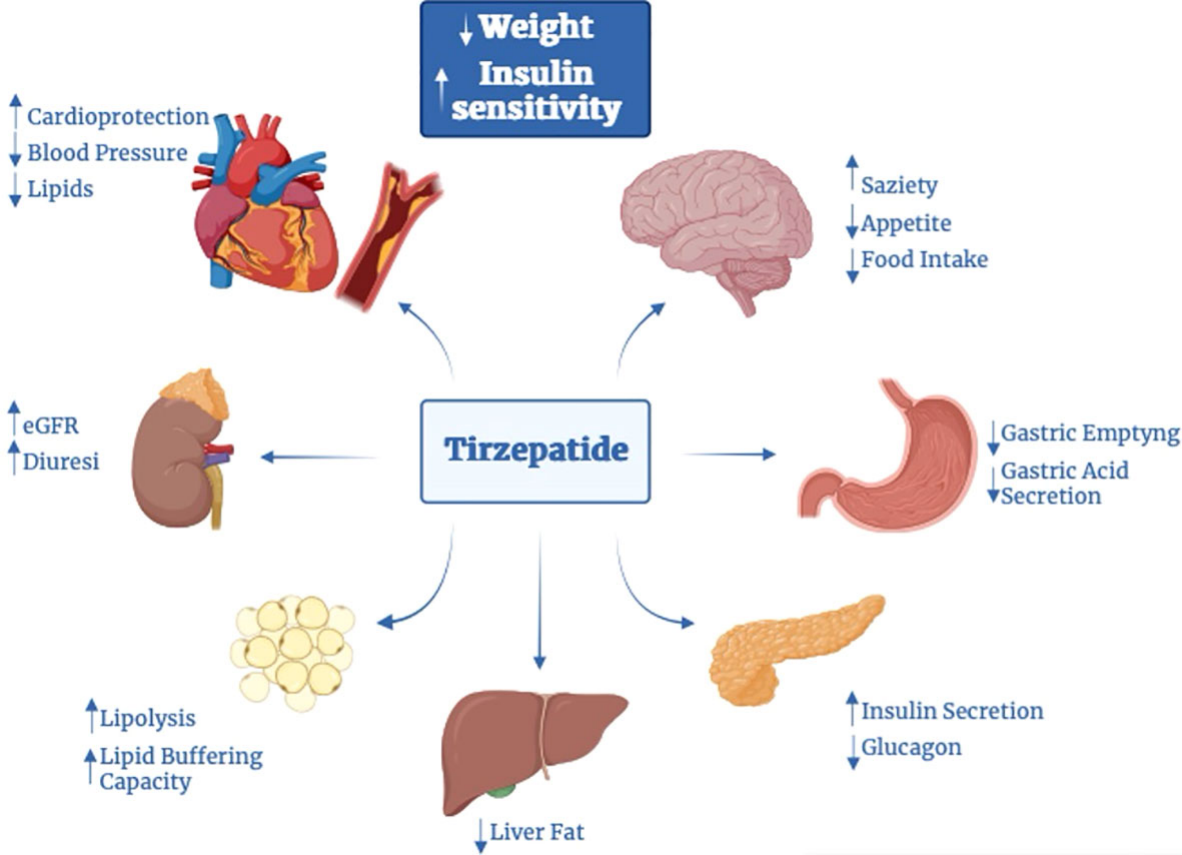
Tirzepatide’s effects on various organs.

On the other hand, in kidneys, the activation of GLP1-R” without receptors, sited in the proximal tubule and pre-glomerular portion, increases cAMP levels, which then triggers PKA activity, inhibiting oxidative renal injury and thus delaying the development of diabetic nephropathy. The action on kidneys appears to be directly through the effects also on renin-secreting cells of the juxta-glomerular apparatus, whose stimulation may lead to increased natriuresis and reduce hyperfiltration, and indirectly by the reduction of angiotensin II levels, the increase in nitric oxide with consequently endothelial vasodilation and the improvement of risk factors of kidney disease (hyperglycemia, hypertension, obesity) (20). In this sense, the role of TZP may also be suggested (**Figure 2**).

## 2 Data of efficacy

### 2.1 Clinical trials: the SURPASS program

**Figure 3:**
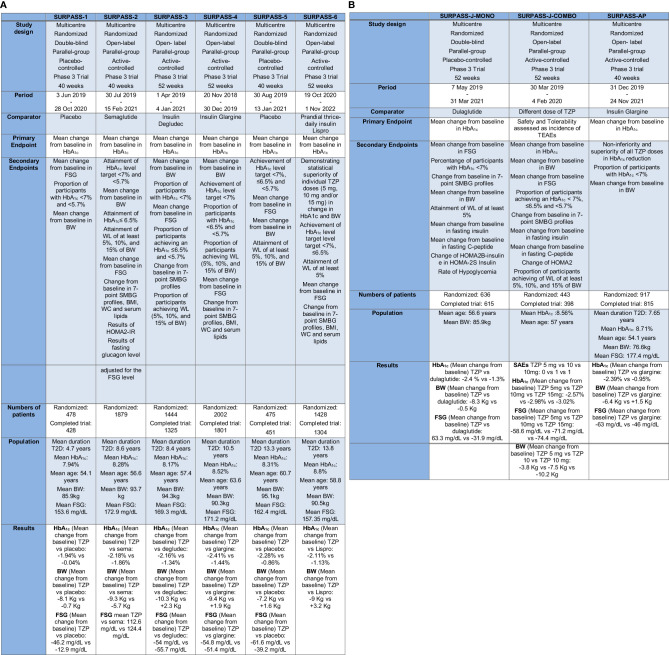
**(A)** The SURPASS Program (SURPASS1–6). TZP, Tirzepatide; FSG, Fasting serum glucose; BW, Body Weight; HbA1c, Glycated Hemoglobin; T2D, Type 2 Diabetes; SMBG, self-monitored blood glucose; WC, waist circumference; HOMA2-IR, homeostasis model assessment–insulin resistance. **(B)** The SURPASS Program (J-mono, J-combo e AP) TZP, Tirzepatide; FSG, Fasting serum glucose; BW, Body Weight; HbA1c, Glycated Hemoglobin; T2D, Type 2 Diabetes; SMBG, self-monitored blood glucose; WC, waist circumference; HOMA2B-Insulin, homeostasis model assessment B–insulin; HOMA2S-Insulin, homeostasis model assessment S–insulin; TEAEs, treatment-emergent adverse events; SAEs, serious Adverse Event(s).

The central clinical trial program designed to demonstrate the therapeutic effectiveness (in terms of GC and BW reduction), tolerability and safety of TZP in diabetic patients was the SURPASS program. It encompasses trials SURPASS from 1 to 6 (**Figure 3A**), SURPASS-J (mono and combo) and SURPASS-AP, performed respectively on Japanese and Asian-Pacific population (**Figure 3B**), and SURPASS CVOT.

All these trials amount standard features: TZP is surveyed at three decisive doses (5, 10, and 15 mg per week); the treatment is started at the dose of 2.5 mg and increased every four weeks. Some SURPASS trials use active comparators like GLP-1RA, or basal insulin preparations, while others compare TZP to placebo.

In the most of the studies the primary endpoint is a change in HbA1c from baseline, except for the SURPASS J combo, where the primary endpoint is the number of patients with more than one critical adverse event (69, 70).

The SURPASS-1 trial studied the efficacy and safety of the three doses of TZP versus placebo in diabetic drug-naïve patients not adequately controlled with a lifestyle mediation (diet and exercise). All the three doses of TZP confirmed an important decrease in HbA_1c_ and BW. It demonstrated to have a safety profile similar to GLP-1RA not increasing the risk of hypoglycemia (19, 48).

In the SURPASS-2 study, TZP at the dose of 5, 10, and 15 mg were compared to the GLP-1RA semaglutide (1 mg once weekly) in patients not well controlled in therapy only with metformin (44, 71). In summary, TZP was greater to semaglutide in reducing HbA_1c_ (31, 44, 71).

The SURPASS-3 study was started to study the efficacy and safety of TZP versus basal insulin degludec in diabetic patients inadequately controlled by metformin with or without an SGLT2-i for 52 weeks (72–74). A sub-study of this trial focused on GC through the use of continuous glucose monitoring (CGM) and the proportion of time within a tight predefined “time in range” (TIR) from 71 to 140 mg/dL at the end of the study was considerably more significant in the group of participants receiving either 10 or 15 mg TZP competed to the group taking insulin degludec (72, 73, 75).

In the 52-week SURPASS-4 trial TZP was tested versus insulin glargine in diabetic adults and a high CV risk below not sufficient GC at baseline and in therapy with one to three oral glucose-lowering drugs (metformin, sulfonylurea, SGLT2-i). In summary, TZP attained more pronounced HbA_1c_ reductions at the end of the study competed to insulin glargine, with also a lower incidence of hypoglycemia. TZP treatment was not associated with increased CV risk (72, 76).

The SURPASS-5 study evaluated the efficacy and safety of an injectable mixture therapy: TZP and insulin glargine. Diabetic patients in therapy with metformin and insulin glargine as baseline therapy and not adequately controlled received either TZP or placebo during the 40-week study span (72, 77). Patients with T2D who received the additional therapy with TZP reached statistically significant improvements in GC after 40 weeks (72).

In the SURPASS-6 trial, TZP was compared to insulin Lispro three times daily in diabetic patients previously treated with a dose of insulin glargine, with or without metformin. Weekly TZP compared with prandial insulin, administered in addition to insulin glargine, proved reductions in HbA_1c_ and BW, not increased the risk of hypoglycemia (78, 79).

The SURPASS-J-mono study was a phase 3 clinical trial performed in Japan. It comprised adults with T2D who had discontinued an oral glucose-lowering medication or were treatment-naïve. TZP was superior to dulaglutide in GC and reduction of BW and the safety report of TZP was coherent with that of dulaglutide (80).

The SURPASS-J-combo trial embraced diabetic adults with HbA_1c_ 7% to 11% and BMI ≥ 23 kg/m^2^, stable weight and uncontrolled with therapy of metformin, thiazolidinedione, sulfonylureas, meglitinide, alpha-glucosidase inhibitor or SGLT2-i (81). TZP was well tolerated as an add-on to oral glucose-lowering drugs monotherapy in Japanese diabetic participants and showed development in GC and BW, irrespective of undercurrent glucose-lowering drugs (82).

In SURPASS-AP trial insulin-naive diabetic patients not adequately controlled on therapy with metformin (with or without a sulphonylurea) in Australia, India, China and South Korea, were randomized to TZP 5 mg, 10 mg or 15 mg or insulin glargine. TZP was generally well tolerated and confirmed greater diminution in HbA_1c_ compared to insulin glargine in an Asia-Pacific patients, in particular in Chinese population with T2D (83).

The SURPASS-CVOT trial, where dulaglutide at the dose of 1.5 mg/week or at the highest tolerated dose, is the comparator, has a distinctive design from the other trials: the primary endpoint is the time to the first manifestation of any major adverse CV event (MACE), defined as myocardial infarction, stroke or CV death. The study is fully recruited and ongoing (84).

HbA_1c_ was reduced in SURPASS 1–5, using from 5 to 15 mg of TZP per week, by between 1.69 to 2.58%, and a new plateau of HbA_1c_ and fasting serum glucose (FSG) was reached with approximately 24–30 weeks of treatment. FSG was reduced in SURPASS 1–5 between 43 and 63 mg/dL, and BW was reduced by between 5.4 to 11.7 kg in SURPASS 1–5. Extraordinarily, a plateau wasn’t achieved in trials with a duration shorter than 52 weeks; to reach a new steady state about BW it may take more than a year after initiating TZP treatment (85).

Furthermore, TZP was significantly more efficacious than titrated basal insulins degludec and glargine (75). In these trials, higher doses of TZP were at least as successful as basal insulin in monitoring FSG.

However, the substantially better efficacy (concerning HbA_1c_ and BW reductions) compared to semaglutide at the standard dose used in most type 2 T2D trials was the most remarkable finding (85, 86). From this treatment difference we can deduce that GIP-R agonism contributes considerably to the global efficacy of TZP.

The relative reductions in HbA_1c_ and BW observed with TZP in all its final doses were comparable among the SURPASS trials. The reduction in HbA_1c_ is independent of age (85), duration of T2D (87), or baseline HbA_1c,_ with meaningful reductions in all of the subgroups, even if the reduction is more significant in patients with higher HbA_1c_ at the baseline.

Concerning BW, a higher baseline BMI predicts absolute weight reduction. However, a substantial body WL is detected even in patients with a BMI <27 kg/m^2^. So, there is no significant difference about sex between females and males in patients treated with TZP.

**Figure 4:**
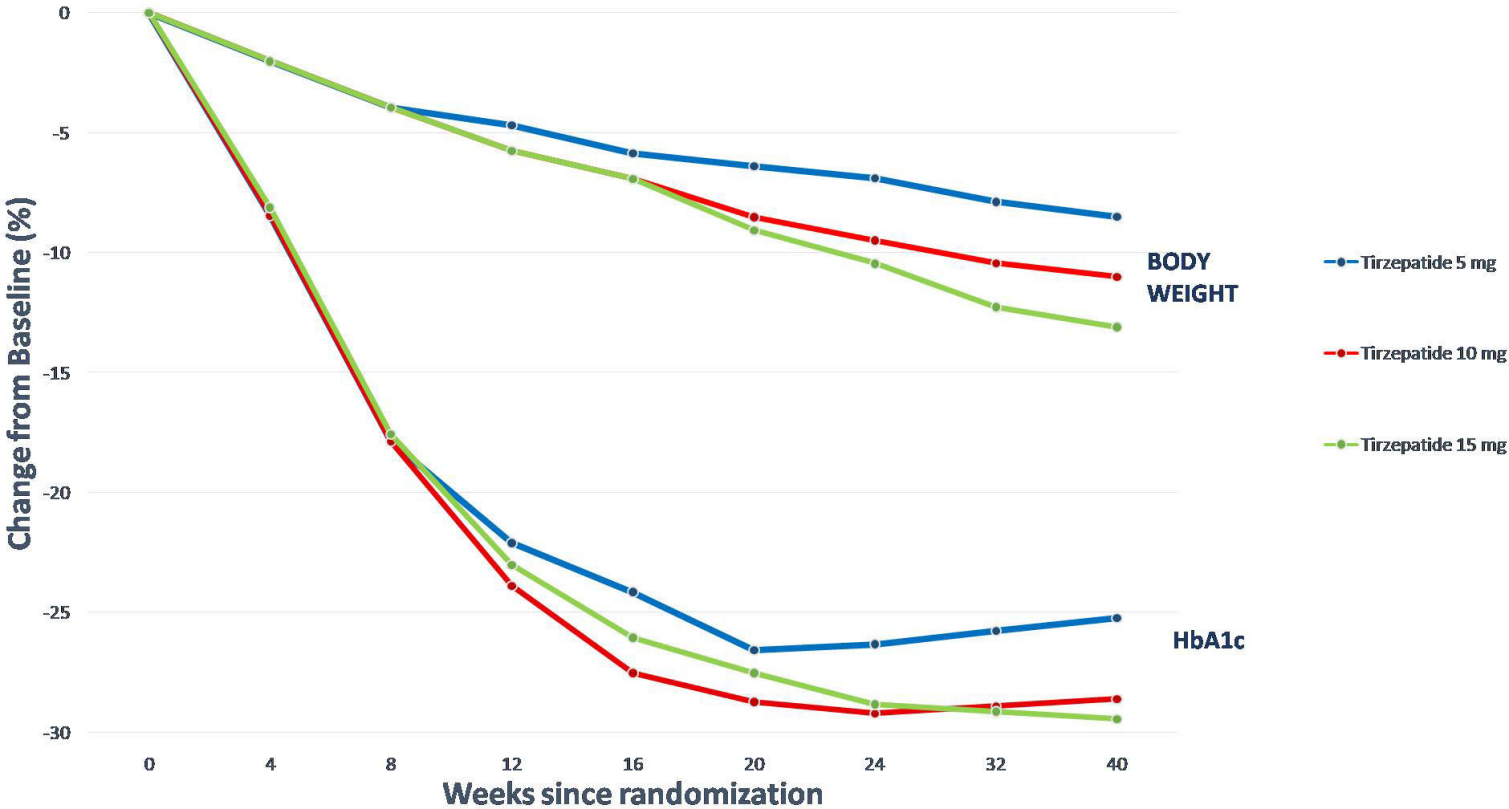
Tirzepatide’s effects on body weight and in HbA_1c_ (SURPASS 2).

Another remarkable finding from the SURPASS trials is that there is a significant relationship between WL and the decrease in HbA_1c_, demonstrating that more significant WL attainable with TZP, has a considerable impact on GC and, accordingly, on the HbA_1c_ (**Figure 4**).

Approximately 38% of patients cared for TZP reached an HbA_1c_ <5.7%, a nondiabetic value. This subgroup was characterized by a shorter duration of T2D, slightly younger age, lower baseline FSG and HbA_1c_, and more significant BW reduction. There wasn’t a difference in the baseline BMI value in patients who didn’t reach an HbA_1c_ <5.7%. Thus, there is a higher likelihood of response in patients with less advanced T2D; there is a substantial inter-individual variability in treatment effects (85).

#### 2.1.1 Effects on lipid structure and on the liver

The SURPASS-2, where TZP (with metformin) was compared to semaglutide (74, 88), showed adjunctive effects, such as lowering the concentrations of LDL and triglycerides and elevating the concentration of HDL.

The main suppliers to the progression of MAFLD are hepatic steatosis and insulin resistance.

Due to the above-mentioned effects of TZD on glycemic, hepatic metabolism, and inflammation, treatment with dual-agonism may reduce or reverse liver damage (lobular inflammation, hepatic steatosis, liver cell damage, and fibrosis) and metabolic dysfunction (35, 41).

GLP-1RA has multiple hepatic effects on MAFLD, involving an adaptation of portal and plasma glucagon and insulin concentrations, hepatic insulin sensitivity and improving hepatocyte mitochondrial function (89) liver enzymes and hepatic fat accumulation (90) and reducing adipose tissue lipotoxicity as well as promoting improvement of steatohepatitis in patients with MAFLD and stimulating WL as well as weight-independent mechanisms (91) (92). The reduction in BW and the improved GC are important factors in reducing NAFLD parameters, reducing hepatic steatosis and fibrosis (93) and improving hepatic necro-inflammation (94, 95). Various mechanisms have been theorized to describe how dual agonism, as GLP-1 RA, could directly reduce triglycerides’ hepatocyte storage, lipogenesis and improving hepatic glucose metabolism (96) and promoting lipolysis and fatty acid oxidation (97). So, it can reduce macrophage infiltration of adipose tissue, inhibit inflammatory pathways in adipocytes and ameliorate insulin sensitivity (98). Compared with GLP1-RA, TZP had more potent hypoglycemic and weight-loss effects (99, 100).

There is a randomized controlled phase 2 study with the objective to explore the use of TZP as a treatment for MAFLD, providing strong evidence [NCT04166773]. However, cornerstones of management are, if needed, promoting a healthy lifestyle and WL. Adherence remains an important challenge, but it may not be sufficient for critical disease activity or advanced fibrosis (101).

### 2.2 SURMOUNT study program

**Figure 5:**
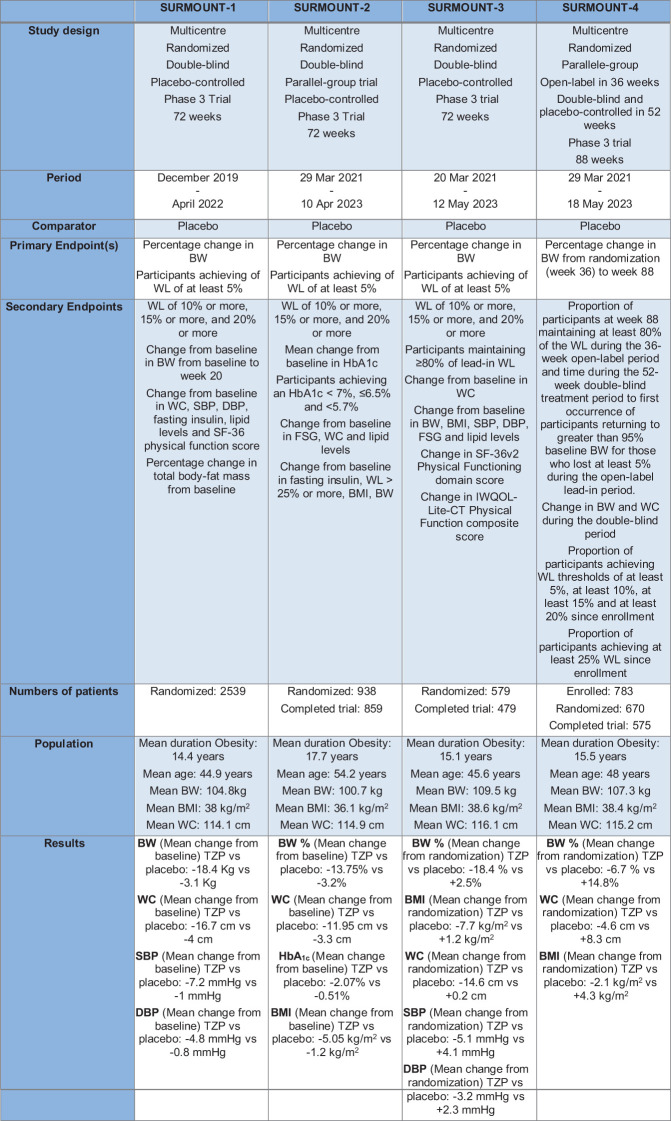
The Surmount Program. TZP, Tirzepatide; BW, Body Weight; WL, Weight Loss; WC, waist circumference; SBP, Systolic Blood Pressure; DBP, Diastolic Blood Pressure.

TZP treatment induced a significant BW reduction in diabetic patients who were obese or overweight. Based on these findings, the SURMOUNT trials program was designed to study TZP’s efficacy and safety in the treatment and management of obesity. It is a clinical trial program that includes randomized controlled studies with a duration of at least 72 weeks. In SURMOUNT-2, SURMOUNT-3 and SURMOUNT-4 were used TZP doses of 10 and 15 mg and all three doses, including the 5-mg dose, were used in SURMOUNT-1. The primary endpoint for all studies is the per cent change from randomization in BW (**Figure 5**). Another study, not yet published, is SURMOUNT-OSA, a clinical trial of 52-weeks that examined the efficacy and safety of TZP versus placebo in obese participants and an established OSA diagnosis for treatment of moderate and severe OSA (102).

The SURMOUNT-1 study was a clinical trial of 72 weeks performed in 2539 obese patients without T2D that were randomized in four arms with placebo or TZP at the dose of 5mg,10 mg or 15 mg (72). TZP treatment was correlated with reduced systolic blood pressure (SBP), waist circumference, plasma lipid concentrations and fasting insulin (103).

The SURMOUNT-2 study is a clinical study that enrolled 938 diabetic patients, receiving 10 mg or 15 mg of TZP or placebo. In this 72-week trial TZP 10 mg and 15 mg determinate a clinically meaningful reduction in BW, with a safety profile comparable to GLP1-RA- based therapies (104).

The SURMOUNT-3 study, after an organized intensive lifestyle intervention of 12-week, is a randomized 72-week clinical trial where patients receive placebo or TZP at the maximally tolerated dose. When administered following an initial 12-week intensive lifestyle intervention, TZP increased the WL (105).

In the end, SURMOUNT-4 is a trial that studies BW preservation in participants with obesity or overweight. The main purpose is to learn more about how TZP maintains WL. The study has two phases: a lead-in phase in which all participants take TZP and a treatment phase in which participants, after randomization, will either continue TZP or switch to placebo. The general mean weight reduction for TZP and placebo from weeks 0 to 88 were respectively 25.3% and 9.9% (106).

### 2.3 Tirzepatide and hypertension: the role of insulin resistance and hyperinsulinemia

Clinical studies showed that concomitant developments in insulin sensitivity, β-cell, and α-cell function underpin the effects of TZP on GC.

Furthermore, clinically meaningful blood pressure reductions were observed in participants receiving TZP. The mean changes in SBP of -2.8 to -12.6 mm Hg and mean reductions in diastolic blood pressure (DBP) of -0.8 to -4.5 mm Hg were reported (107).

The factors behind obesity-induced hypertension are numerous, and often, they are effective concurrently. They include changes in the production of constricting and relaxing factors endothelium-derived, interruption of molecular signaling, increased oxidative stress, renal injury, insulin resistance, hyperinsulinemia, sleep apnea syndrome, that make hemodynamic alterations (108, 109). Also, adipose tissue provides to determine endothelial dysfunction by secreting adipokines (109–111).

Insulin resistance and hyperinsulinemia are often present in obese individuals and play a significant role in the genesis of hypertension (112, 113). Some studies have evidenced that hyperinsulinemia may activate the renin-angiotensin system, increase sympathetic nervous system activity and renal sodium retention and, if sustained, all these elements could increase blood pressure (112, 114). Insulin can activate transporters such as the Sodium-proton exchanger type 3 (NHE3) in the proximal tubule and the epithelial sodium channel (ENaC) in the distal nephron and in the connecting tubule, another vital contributor to sodium reabsorption (112, 115). In addition, insulin regulates w-no-lysine (WNK) kinases, which, through the stimulation of sodium reabsorption in the distal nephron, are responsible for familial hypertension (112, 116).

In the SURPASS-1 trial, TZP 10 mg resulted in a mean decrease in SBP of 5.2 mmHg, which is more significant than that with a placebo. Likewise, in the SURPASS-3 trial, all three doses of TZP caused a significant decrease in mean SBP from baseline (from 4.9 to 6.6 mmHg).

We can use SURMOUNT 3 as a reference within the surmount program to confirm the effectiveness of TZP in improving tension values.

**Figure 6:**
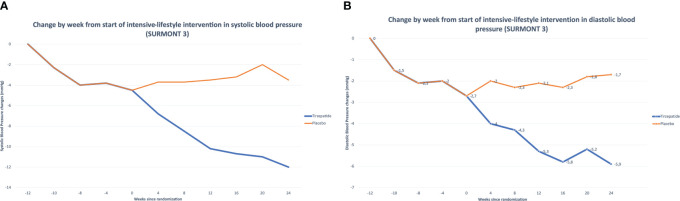
**(A)** Tirzepatide’s Effects on Systolic Blood Pressure (SURMONT 3). **(B)** Tirzepatide’s Effects on Diastolic Blood Pressure (SURMONT 3).

In this trial, from randomization to week 72, TZP determinates more significant improvements versus placebo in both SBP (TZP −5.1 mmHg, placebo, 4.1 mmHg (**Figure 6A**) and DBP (TZP −3.2 mmHg, placebo 2.3 mmHg) (105) (**Figure 6B**).

### 2.4 Other clinical trials of Tirzepatide’s efficacy

Recently, in addition to the clinical trials mentioned above, which have given impressive results, a trial based on SURPASS-2 study’s general protocol was recently stated, including physiological outcomes. It’s a multicenter, double-blind, randomized, phase 1 study, edited by Heise et al. to estimate insulin sensitivity in patients treated with placebo, TZP or semaglutide after 28 weeks of treatment, achieving a hyperinsulinemic, euglycemic clamp experiment, followed by a hyperglycemic clamp (216 mg/dL) to estimate insulin secretory responses. On the second day, glycemia, glucagon and insulin responses were documented afterwards a mixed meal test with a concurrent valuation of ratings of hunger, satiety and prospective food consumption; on the occasion of an ad libitum meal, energy intake was measured.

The clinical outcomes imitated the same findings of SURPASS-2 in terms of HbA_1c_ reduction and WL. With TZP treatment, insulin sensitivity rises by 65.7%, measured by the glucose infusion rate indispensable to maintain euglycemia, and the rise was 20.5% greater with TZP than with semaglutide 37.5% with semaglutide 1 mg). Some of this effect is likely due to the change in WL (6.9 kg with semaglutide versus 11.2 kg with TZP).

There may be weight-independent and weight-dependent factors associated with TZP treatment that improve insulin sensitivity and a more substantial improvement in insulin sensitivity with TZP compared to semaglutide per unit WL.

There was also a significant reduction in fasting and post-meal glycemia with both semaglutide and TZP compared with placebo, with no difference at the baseline-subtracted plasma glucose between TZP and semaglutide.

Meal-related insulin secretory responses were meaningfully higher with semaglutide and placebo than TZP treatment. These findings help to explain some aspects of TZP’s clinical efficacy, but from this experiment, it’s impossible to discriminate whether the different effects of TZP are the result of GIPR signaling (85, 117, 118).

In another Clinical Trial, Thomas et al. showed that TZP in diabetic patients resulted in substantially greater GC and WL versus dulaglutide. This is a *post hoc* exploratory biomarker study that explored the results of TZP on insulin sensitivity and pancreatic β-cell function. The results are that β-cell function was improved, and fasting glucagon levels were reduced in patients treated with TZP, better than dulaglutide, improving several markers of pancreatic β-cell function, as exposed by dose-dependently decreasing proinsulin levels, proinsulin/insulin ratios and proinsulin/C-peptide ratios and increasing HOMA2-B indices. These data may be suggestive of developments in pancreatic β-cell function because increased circulating proinsulin/insulin ratios and proinsulin/C-peptide are important markers of initial and increasing pancreatic β-cell secretory dysfunction.

HOMA2-IR indices of insulin resistance and fasting insulin levels was reduced by TZP treatment. TZP’s IS effects were only partially attributable to WL, recommending that dual agonism gives distinct instruments of GC (119).

## 3 Conclusion

The troubling rise in the number of subjects affected by obesity worldwide has necessitated new scientific developments to blunt previous adverse effects of medication and facilitate administration, addressing different problems with a single drug. TZP has shown encouraging results in WL, high blood pressure, and HbA_1c_. Patient compliance is encouraged since it has the vantage of a once-week dosing. In this sense, TZP could represent a breakthrough due to its magnitude of effects on WL, blood pressure, and glycemia. It also opens a new scenario in obesity treatment with the promise of finally being the silver bullet against obesity.

## Author contributions

SC: Writing – review & editing, Writing – original draft, Validation, Supervision, Methodology, Conceptualization. CP: Writing – original draft, Investigation, Formal analysis, Data curation. DM: Writing – original draft, Investigation, Formal analysis, Data curation. AT: Writing – original draft, Investigation, Formal analysis, Data curation. CA: Writing – review & editing, Writing – original draft, Validation, Supervision, Methodology, Conceptualization.
